# Development of an enzyme-linked immunosorbent assay using recombinant protein antigen for the diagnosis of Chikungunya virus

**DOI:** 10.1016/j.dib.2019.104015

**Published:** 2019-05-23

**Authors:** Flávia Fonseca Bagno, Lara Carvalho Godoi, Natalia Salazar, Glauco de Carvalho Pereira, Maria Marta Figueiredo, Flávio Guimarães da Fonseca

**Affiliations:** a Departamento de Microbiologia, Universidade Federal de Minas Gerais, Belo Horizonte, Brazil; b Centro de Tecnologia de Vacinas/UFMG, Belo Horizonte, Brazil; c Colégio Técnico da Universidade Federal de Minas Gerais, Belo Horizonte, Brazil; d Fundação Ezequiel Dias (FUNED), Belo Horizonte, Brazil

**Keywords:** *Chikungunya virus*, Diagnostics, ELISA, E2 Envelope protein, Recombinant protein

## Abstract

We describe here the development of an in-house enzyme linked immunosorbent assay (ELISA) for the diagnostic of *Chikungunya virus* (CHIKV) infections using a recombinant protein from CHIKV. The recombinant protein gene was designed based on 154 sequences and we used computational methods to predict its structure and antigenic potential. To confirm predictions, the gene coding for the recombinant CHIKV protein (rCHIKVp) was synthetized and expressed in prokaryotic system. Subsequently, the protein was purified by affinity chromatography and used as antigen in an indirect ELISA. We present data regarding the optimization of the recombinant antigen production and preparation of the ELISA to detect IgG against CHIKV in human sera.

**Table:** Specifications Table

Subject area	*Virology*
More specific subject area	*Virus diagnostics*
Type of data	*One Table and two graphs (Figures)*
How data was acquired	*Data were basically acquired by spectrophotometry using an ELISA plate reader - Multiskan™ GO Microplate Spectrophotometer (Thermo Fisher Scientific, USA). Recombinant proteins were obtained using ÄKTAprime plus (GE Healthcare, USA)*
Data format	*Analyzed data are shown*
Experimental factors	*Samples are crude human sera that were simply heated to eliminate complement factors. The ELISA's recombinant antigen was obtained by affinity chromatography and maintained in 6 M urea up to use.*
Experimental features	*The ELISA's recombinant antigen - the E2 protein from Chikungunya virus - was expressed in E. coli transformed cells and purified by affinity chromatography. The purified protein was used as the antigenic solid phase of the ELISA and was tested against a bank of characterized human sera, composed of Chikungunya-positive sera, Dengue-positive sera or Zika-positive sera. Sera that are negative for all viruses were also used.*
Data source location	*Minas Gerais State, Brazil.*
Data accessibility	*State if data is with this article.*
Related research article	*This data article is submitted as a companion paper to:* *Bagno, FF, Figueiredo, MM, Villarreal J, Pereira, GC, Godoi, LC, da Fonseca, FG. Undetected Chikungunya Virus Co-Infections in a Brazilian Region Presenting Hyper-Endemic Circulation of Dengue and Zika. J. Clin. Virol. In press.*

**Table:** 

**Value of the Data** • This data provides details about the design and production of a recombinant Chikungunya virus protein (rCHIKp). • We describe the standardization of an in house ELISA using the rCHIKp as an antigen. • The developed ELISA presented the ability to distinguish chikungunya-positive human sera from virus-negative sera. • The ELISA was also able to efficiently distinguish patients that were seropositive for chikungunya from those that were seropositive for dengue or zika.

## 1 Data

**Fig. 1:**
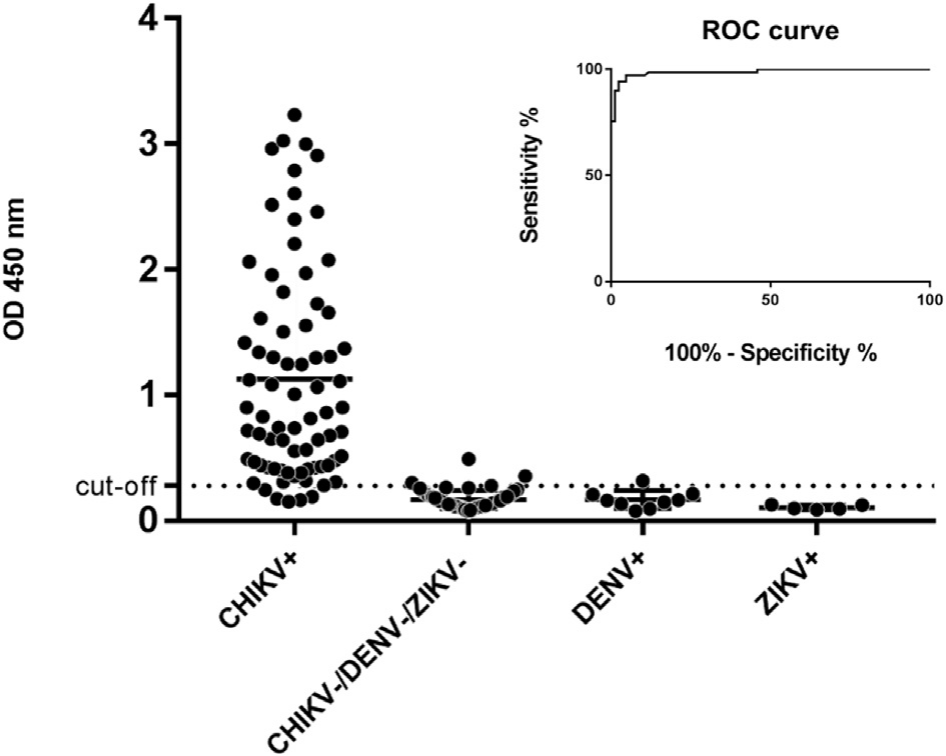
Seroreactivity of the rCHIKVp using the developed indirect ELISA against Chikungunya-positive sera (CHIKV+) and virus-negative sera (CHIKV-/DENV-/ZIKV-). Potential cross reactivity with sera samples that were positive for co-circulating arboviruses were also tested: Dengue positive (DENV+) and Zika positive (ZIKV+) sera. A cut-off value of 0,279 was obtained, according to the Roc curve (inset).

We design a synthetic gene coding for a recombinant *Chikungunya virus* protein (rCHIKp) to be used as an antigen in serologic assays. *In silico* analysis revealed that rCHIKp secondary structure has predominant coils and identified B lymphocyte epitopes in regions of structural disorder, which are advantageous for antigenic recognition. The overall antigenic prediction score was 0.53 (which suggests a probable efficient antigen). The gene was expressed in *E. coli* cells and purified by affinity chromatography. Coomassie Brilliant Blue staining showed the presence of rCHIKp with the predicted molecular mass (_∼_42 kDa). Next, we developed an In House Enzyme-Linked Immunosorbent Assay (ELISA) for the detection of immunoglobulin G (IgG) anti-CHIKV using the rCHIKp as the ELISA's antigenic solid phase. The rCHIKVp-based ELISA showed a sensitivity value of 95% and specificity of 96% (Table 1). No cross-reactivity was found against sera from Zika- (ZIKV) and Dengue- (DENV) positive patients (Fig. 1). The developed ELISA was used in the assessment of human patients suspected of arboviral infections in Minas Gerais State, Brazil [1].

**Table 1:** Relative sensitivity and specificity of the rCHIKVp-based in house-Anti-CHIKV ELISA in comparison to a commercial kit (*Chikungunya IgG ELISA Euroimmun, Germany*).

		In house-Anti-CHIKV ELISA		
		No. of pos.	No. of neg.	Parameters
Commercial kit	Pos. (n = 71)	68	3	Sensitivity: 96%
	Neg. (n = 76)	74	2	Specificity: 97%

## 2 Experimental design, materials, and methods

### 2.1 Design of the antigen gene

CHIKV genome sequences deposited at GenBank were aligned using MEGA7 software [2]. The selection criteria were: complete annotation of the genome and absence of indefinite nucleotides in the sequence. Brazilian (n = 42) and foreign samples belonging to the genotypes circulating in Brazil were used. Sequences belonged to the Asian genotype (n = 112) and East-Central South African (ECSA) genotype (n = 274). A consensus sequence was generated for each group (Group 1: Brazilian samples, Group 2: Asian genotype samples and Group 3: ECSA genotype samples). These consensual sequences were, then, compared to each other to generate a unique nucleotide sequence.

The gene sequence was codon-optimized for expression in *E. coli* by OptimumGene ™ - Codon Optimization software (Genescript). Transmembrane and hydrophobicity analyzes were performed using TMHMM (http://www.cbs.dtu.dk/services/TMHMM/) [3] and Protscale -Expasy (http://web.expasy.org/Protscale/) [4]. From these predictions, a cut-off point for the generation of the protein without its transmembrane domain was determined. The nucleotide sequence of the truncated protein-coding gene was commercially synthesized and subcloned into the pET-21 expression vector, which included a histidine tag to the construction.

### 2.2 In silico analysis

To evaluate the potential of our rCHIKVp as a diagnostic tool, the protein's predicted amino acid sequence was submitted to BepiPred (http://www.cbs.dtu.dk/services/BepiPred/) for the prediction of linear B cell epitopes [5] and IUPred (http://iupred.enzim.hu/), for the prediction of intrinsic structural disorder, indicating absence of secondary structure and, consequently, regions of possible interaction with antibodies [6]. The antigenicity of the designed protein was evaluated by the VexiJen V2.0 online server (http://www.ddphpharmfac.net/vaxijen/VaxiJen/VaxiJen.html) [7].

### 2.3 Recombinant protein production

The pET-21 vector containing the gene of interest was used to transform *E. coli* BL21(DE3) strain by heat shock. Plasmid-positive clones were induced by IPTG and expression of the recombinant protein was optimized and analyzed by SDS-PAGE. The antigen was purified by affinity chromatography using nickel columns in an *ÄKTAprime plus system (GE Healthcare, USA)*.

### 2.4 In house *Anti*-Chikungunya virus ELISA

The seroreactivity of the rCHIKVp was evaluated using a panel of sera samples from human patients, CHIKV seropositive or not, by an in-house indirect IgG ELISA. Strips of polystyrene microwells (Costar, USA) were coated overnight at 4 °C with 100 μl per well of rCHIKVp diluted in carbonate buffer (0.05 M, pH 9.6). Wells were washed five times with phosphate-buffered saline (PBS) containing 0.1% Tween20 (PBS-T) and blocked with 1% bovine serum albumin (BSA, SIGMA,USA) for 2 h at 25 °C. Then 100 μl of serum diluted in PBS-T was added and incubated for 1 hour at 37 °C. After five washes, we added 100 μl of horseradish peroxidase (HRP)-conjugated anti-human IgG goat immunoglobulin (Fapon, China), diluted at 1:100.000 in stabilizing diluent (MOSS, USA). Plates were incubated for 30 min at 37 °C, washed five times, and 100 μl of TMB (3,3′, 5,5; -tetramethylbenzidine, MOSS, USA) were added and incubated for further 15 min. Then, 100 μl of H_2_SO_4 ,_ 0,5 M solution was add to the wells to stop the reaction. The plates were analyzed in a Microplate Reader at an optical density (O.D.) of 450 nm.

**Fig. 2:**
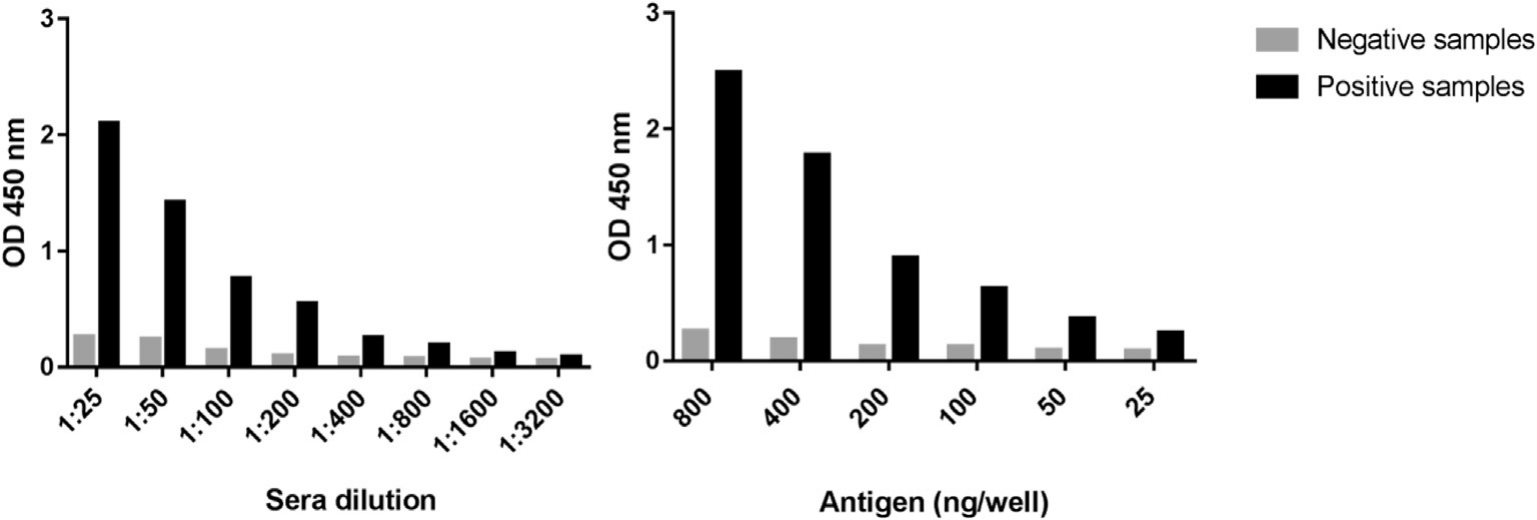
Standardization of in house anti-CHIKV ELISA IgG, with determination of the optimal sera dilution and antigen concentration.

The optimal concentration of the recombinant rCHIKVp per plate well was determined based on a clear distinction of anti-CHIKV antibodies using positive and negative samples. We tested a range of 25–800 ng/well of rCHIKVp and sera dilutions ranging from 1:25 to 1:3200 (Fig. 2). The cut-off value was determined by ROC curve analysis, and an index (*I*) of each absorbance value of the patient sample over the value of cut-off was calculated, according to the equation:

*I* = *a*/*c*, where *a* is the absorbance of the patient sample and *c* is the cut-off value (0,279).

The data is classified as follows:

*I* < 0.9: negative
0.9 ≤ *I* < 1.1: borderline
*I* ≥ 1.1: positive
